# Supplementary material for: scDRP: disentangled representation learning for predicting single-cell responses to perturbations and estimating individual treatment effects
Source: Brief Bioinform. 2026 Oct 26;27(5):bbag482. doi: 10.1093/bib/bbag482 (PMC13704780; doi:10.1093/bib/bbag482)
Supplement: Supplementary file 1 [file supplementary_materials_bbag482.pdf]

Supplementary Materials

Supplementary Notes

SN1 Identifiability theory of non-linear disentanglement

1.1 General Theory

Recent advances in the theory of disentangled and nonlinear ICA models have progressively relaxed the conditions required for identifiability, i.e., the ability to recover unique latent variables up to subgroup or component-wise invertible transformations and permutations.

Zheng et al. [1] first established Structural Sparsity as a sufficient condition for identifiability in fully unsupervised nonlinear ICA, showing that when each latent factor uniquely determines at least one subset of observed variables (i.e., the intersection of their parents identifies that source), the true latent components can be recovered without any auxiliary variables.

Building upon this, Zheng and Zhang [2] generalized the theory to more realistic settings by introducing partial sparsity, undercompleteness (more observed than latent variables), and partial source dependence. They proved that if structural sparsity and independence hold for only a subset of sources, those sources remain component-wise identifiable, while the remaining (possibly dependent) ones are identifiable up to a subspace. The framework also allows auxiliary variables with only a small number of distinct values to assist identifiability, extending nonlinear ICA to mixed or grouped latent structures.

Most recently, Li et al. [3] unified two previously separate lines of identifiability research, i.e., sufficient distributional changes across auxiliary variables and sparse mixing structures, into a single complementary framework. They demonstrated that sparsity in the mixing process can compensate for insufficient domain variation, while diverse conditional distributions can mitigate violations of sparsity. Under smoothness, conditional independence, and either moderate distributional changes or partial sparsity, both subspace- and component-wise identifiability can be achieved with milder assumptions than required by classical nonlinear ICA results.

These works broaden the identifiable regimes for disentangled representation learning, where the theoretical insights are particularly relevant to single-cell generative modeling, where identifying biologically meaningful latent factors, such as cell identity or state, is paramount. In practice, the structural sparsity corresponds to the modular nature of gene regulatory networks, where each latent factor (e.g., a specific transcription factor) regulates only a sparse

subset of marker genes. Simultaneously, the auxiliary variables manifest as experimental metadata, such as perturbation conditions (e.g., drug treatment or gene knockdown). By leveraging the sparse connectivity of gene modules alongside the distributional shifts induced by these perturbations, generative models can achieve robust identifiability, effectively disentangling intrinsic cellular programs from extrinsic experimental effects.

1.2 Identifiability under noisy observations

While the aforementioned theories primarily focus on deterministic or noiseless mixing processes, real-world generative mechanisms are inevitably corrupted by observation noise, which fundamentally alters the identifiability landscape. Khemakhem et al. [4] provided the rigorous identifiability results for deep latent-variable models under additive noise by embedding nonlinear ICA theory into the variational autoencoder framework. Specifically, for a noisy generative process of the form $x = f(z) + \varepsilon$, where f is an injective nonlinear function and ε denotes independent noise, we introduced a conditionally factorized latent prior $p(z \mid u)$ whose parameters vary with an auxiliary observed variable u. By modeling each latent component with an exponential family whose sufficient statistics are modulated by u, the joint distribution $p(x, z \mid u)$ becomes identifiable up to component-wise invertible transformations and permutations, even in the presence of noise and under undercomplete settings. This result demonstrates that distributional variation across auxiliary variables can overcome the fundamental non-identifiability induced by nonlinear mixing and observation noise, extending classical nonlinear ICA identifiability to realistic stochastic generative models. Importantly, their theory establishes consistency of VAE-based maximum likelihood estimation, showing that both latent variables and generative mechanisms can be recovered (up to trivial indeterminacies) rather than merely matching the marginal data distribution.

Classical structural sparsity-based identifiability results enforce sparsity in the Jacobian of the mixing function to restrict the dependency graph between latent and observed variables. However, explicitly computing and regularizing the Jacobian becomes prohibitively expensive in deep generative models. Instead, we adopt a complementary surrogate by constraining the effective latent dimensionality through learnable gating variables with ℓ_0 regularization. This mechanism encourages only a small subset of latent components to actively participate in the generative process, thereby implicitly inducing sparse dependency structures between latent and observed variables. From a functional perspective, restricting latent participation reduces the expressiveness of admissible mixing functions and limits the class of nonlinear reparameterizations responsible for non-identifiability. While not equivalent to explicit Jacobian sparsity, this latent selection strategy serves as a practical approximation that promotes structural sparsity in deep models with significantly lower computational overhead.

1.3 Regularization on sparsity

Classical structural sparsity-based identifiability results enforce sparsity in the Jacobian of the mixing function to restrict the dependency graph between latent and observed variables. However, explicitly computing and regularizing the Jacobian becomes prohibitively expensive in deep generative models. Instead, we adopt a complementary surrogate by constraining the effective latent dimensionality through learnable gating mechanism $\tilde{z} = g \odot z,\ g \in \{0,1\}^n$ with ℓ_0 regularization [5]. This mechanism encourages only a small subset of latent components to actively participate in the generative process, thereby implicitly inducing sparse dependency structures between latent and observed variables. Moreover, by encouraging only a small number of active gates, the model automatically infers the effective latent dimensionality, mitigating the overparameterization that otherwise exacerbates non-identifiability in nonlinear generative models. Though slightly stronger than classical entry-wise Jacobian sparsity, this latent selection strategy serves as a commonly used and practical approximation that promotes structural sparsity in deep models with significantly lower computational overhead [6].

SN2 Conditional optimal transport on disentangled latent space.

Recall that we assume that the observed variables $\boldsymbol{x}$ are generated by both domain (perturbation)-dependent latent factors $\boldsymbol{z}_d \sim p_d(\boldsymbol{z}_d|\boldsymbol{a},\boldsymbol{w})$ and domain-invariant latent factors $\boldsymbol{z}_u \sim p_u(\boldsymbol{z}_u|\boldsymbol{w})$, where $\boldsymbol{a}$ stands for the domain index, $\boldsymbol{w}$ stands for the confounding, and we assume that $\boldsymbol{z}_d \perp \boldsymbol{z}_u|\boldsymbol{w}$.

The idea of counterfactual matching in the disentangled latent space is that, we assume that the effect of changing domains (perturbations) $\boldsymbol{a}$ on $\boldsymbol{z}_d$ is rank-preserving, conditional on confoundings. That's to say, perturbations might have heterogeneous effects on the latent factors of cells belonging to different cell types or cell states. However, after controlling for these confounding factors (cell type or cell state), the effect of the perturbation on the cells within that subgroup should be similar, meaning that the relative positions (distribution rank or quantile) of these cells before and after the perturbation should be preserved.

We resort to optimal transport (OT) for the implementation of quantile matching, i.e.,

$$\Gamma^* = \mathrm{argmin}_\Gamma \int \left|\left|\boldsymbol{z}_d^s - \boldsymbol{z}_d^t\right|\right|^2 d\Gamma(\boldsymbol{z}^s, \boldsymbol{z}^t),$$

since there is an equivalence between OT with a strictly convex cost (like square Euclidean distance) and quantile matching in one-dimension [7, 8]. In multi-dimensional latent space, although inexact, we can view this as a trade-off in quantile matching across different dimensions, because the squared Euclidean distance can be seen as the summation of the squared Euclidean distances in each dimension.

When confounding $\boldsymbol{w}$ is not observed or not fully observed (e.g. only known a surrogate like cell type labels), $\boldsymbol{z}_u$ can serve as a representation of confounding. It is hard to impose hard conditionals on $\boldsymbol{z}_u$, i.e.,

$$\Gamma^* = \text{argmin}_\Gamma \int \left|\left|\boldsymbol{z}_d^s - \boldsymbol{z}_d^t\right|\right|^2 d\Gamma(\boldsymbol{z}^s, \boldsymbol{z}^t),$$

$$\text{s.t.}\, \boldsymbol{z}_u^s = \boldsymbol{z}_u^t,$$

and we relax to a soft version,

$$\Gamma^* = \text{argmin}_\Gamma \int \left|\left|\boldsymbol{z}_d^s - \boldsymbol{z}_d^t\right|\right|^2 d\Gamma(\boldsymbol{z}^s, \boldsymbol{z}^t),$$

$$\text{s.t.}\, \left|\left|\boldsymbol{z}_u^s - \boldsymbol{z}_u^t\right|\right|^2 \leq \varepsilon.$$

and this is equivalent to solving

$$\Gamma^* = \text{argmin}_\Gamma \int \left(\alpha \left|\left|\boldsymbol{z}_u^s - \boldsymbol{z}_u^t\right|\right|^2 + \beta \left|\left|\boldsymbol{z}_d^s - \boldsymbol{z}_d^t\right|\right|^2\right) d\Gamma(\boldsymbol{z}^s, \boldsymbol{z}^t).$$

We can then generate counterfactual latent factors by barycentric projections on the full latent space

$$\tilde{\boldsymbol{z}}_i^{s\to t} = \frac{\sum_{j=1}^m \Gamma_{ij} \boldsymbol{z}_j^t}{\sum_{j=1}^m \Gamma_{ij}},$$

or on the $\boldsymbol{z}_d$ space only

$$\tilde{\boldsymbol{z}}_i^{s\to t} = \left(\boldsymbol{z}_{u,i}^s, \frac{\sum_{j=1}^m \Gamma_{ij} \boldsymbol{z}_{d,j}^t}{\sum_{j=1}^m \Gamma_{ij}}\right).$$

SN3 Toy examples to illustrate the idea of conditional optimal transport on disentangled latent space.

We constructed synthetic datasets, $\boldsymbol{X}_s$ (Source) and $\boldsymbol{X}_t$ (Target), in a latent space. Each data point $\boldsymbol{x} = [\boldsymbol{z}_u, \boldsymbol{z}_d]^T$ is composed of an **invariant component** ($\boldsymbol{z}_u$) and a **domain-specific component** ($\boldsymbol{z}_d$).

The $\boldsymbol{z}_u$ component is designed to represent conserved cell identity (e.g., cell types), featuring two distinct, well-separated clusters, symmetrically centered around ± 2, ensuring that this fundamental structure is shared between $\boldsymbol{X}_s$ and $\boldsymbol{X}_t$. In contrast, the $\boldsymbol{z}_d$ component models the domain shift or perturbation effect. While the source $\boldsymbol{z}_d$ is centered at 0, the target $\boldsymbol{z}_d$ is generated with cluster-specific shifts (± 2), resulting in a clear domain

separation that is dependent on the underlying $\boldsymbol{z}_u$ identity.

Specifically, the distributions are formulated as:

1. Invariant Factors ($\boldsymbol{z}_u$):

$$z_{u,s}, z_{u,t} \sim Mixture\big(Dist_u(-2,0.25), Dist_u(2,0.5)\big)$$

2. Domain-Specific Factors ($\boldsymbol{z}_d$):

$$z_{d,s} \sim Dist_d(0,1)$$

$$z_{d,t} \sim Mixture\big(Dist_d(2,1), Dist_d(-2,1)\big)$$

By allowing $Dist_u$ and $Dist_d$ to be selected from distributions such as Normal, Laplace, or Uniform, we can explore the robustness of the conditional optimal transport under various distributional differences, ensuring the generated data faithfully captures the need to align $\boldsymbol{z}_d$ while strictly preserving the $\boldsymbol{z}_u$ clustering structure.

We first conducted simulations in a setting where both $\boldsymbol{z}_d$ and $\boldsymbol{z}_u$ are 1-dimensional, and presented the scatter plots of the counterfactual generations $\boldsymbol{x}$ (Supplementary Fig.S1) and the marginal distributions of $\boldsymbol{z}_d$ and $\boldsymbol{z}_u$ (Supplementary Fig.S2). It can be seen that when no conditioning is performed ($\alpha = 0$) or a very small α is set, significant cross-matching occurs between different groups. Although the marginals can be aligned, there is a clear issue of confounding interference. The issue becomes even more severe when performing barycentric mapping on $\boldsymbol{z}_d$ only, as the lack of conditioning leads to a more severe distribution shift. Conversely, when $\boldsymbol{z}_u$ is fully utilized for confounding matching without $\boldsymbol{z}_d$ transfer ($\beta = 0$), we observe that while the marginals of $\boldsymbol{z}_u$ can be aligned, the marginals of $\boldsymbol{z}_d$ exhibit significant differences. The simulations conducted on a multi-dimensional latent space further corroborate this point (Supplementary Fig.S3). Therefore, using an appropriate level of conditioning is crucial.

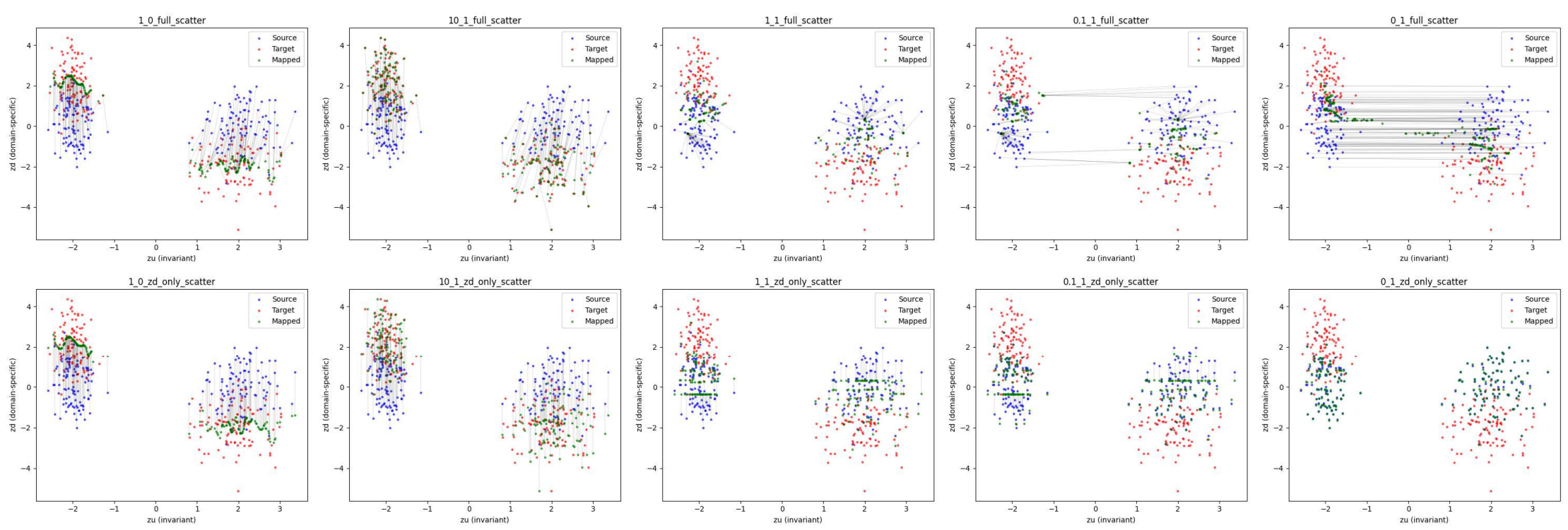

Supplementary Fig.S1 The scatter plots of the counterfactual generations in 1-dimensional latent-space toy example. The title of each subplot shows the configuration

(alpha_beta_projection method) used.

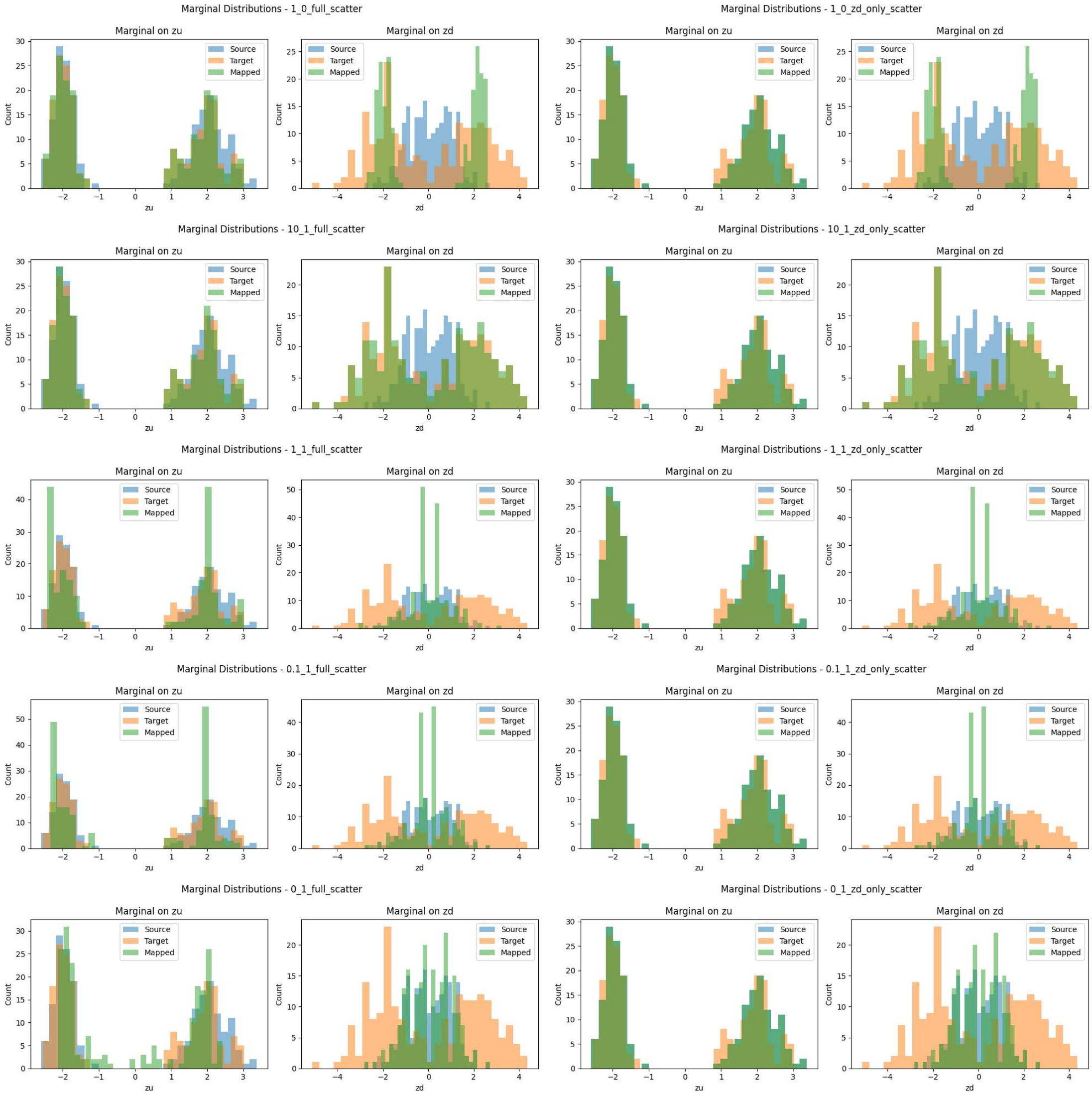

Supplementary Fig.S2 The marginal distributions of $\boldsymbol{z}_d$ and $\boldsymbol{z}_u$ in 1-dimensional latent-space toy example. The title of each subplot shows the configuration (alpha_beta_projection method) used.

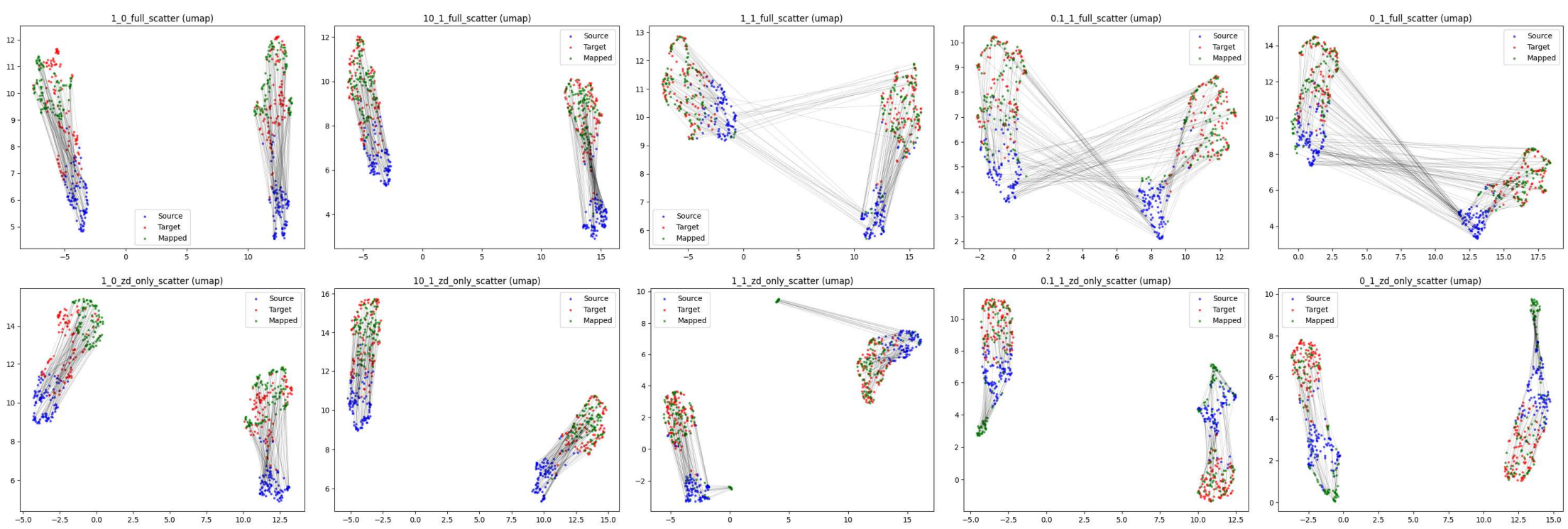

Supplementary Fig.S3 The scatter plots of the counterfactual generations in multi-

dimensional latent-space toy example ($dim(\boldsymbol{z}_d) = dim(\boldsymbol{z}_u) = 10$ here). The title of each subplot shows the configuration (alpha_beta_projection method) used.

Reference:

[1] Zheng, Y., Ng, I., & Zhang, K. (2022). On the identifiability of nonlinear ICA: Sparsity and beyond. *Advances in neural information processing systems*, *35*, 16411-16422.

[2] Zheng, Y., & Zhang, K. (2023). Generalizing nonlinear ICA beyond structural sparsity. *Advances in Neural Information Processing Systems*, *36*, 13326-13355.

[3] Li, Z., Fan, S., Zheng, Y., Ng, I., Xie, S., Chen, G., ... & Zhang, K. (2025). Synergy Between Sufficient Changes and Sparse Mixing Procedure for Disentangled Representation Learning. In *The Thirteenth International Conference on Learning Representations*.

[4] Khemakhem, I., Kingma, D., Monti, R., & Hyvarinen, A. (2020). Variational autoencoders and nonlinear ica: A unifying framework. In *International conference on artificial intelligence and statistics* (pp. 2207-2217). PMLR.

[5] Louizos, C., Welling, M., & Kingma, D. P. (2018). Learning Sparse Neural Networks through L0 Regularization. In *International Conference on Learning Representations*.

[6] Xie, S., Kong, L., Gong, M., & Zhang, K. (2023). Multi-domain image generation and translation with identifiability guarantees. In *The Eleventh international conference on learning representations*.

[7] Chewi, S., Niles-Weed, J., & Rigollet, P. (2024). Statistical optimal transport. *Springer*.

[8] Balakrishnan, S., Kennedy, E., & Wasserman, L. (2025). Conservative inference for counterfactuals. *Journal of Causal Inference*, *13*(1), 20230071.

SN4 Validating the control-to-treatment conditional rank-preserving correspondence.

Design. Following Methods Section 4.6, we simulated a small-size example data with 200 genes, in which each treated cell is generated as a constant, cell-type-specific additive shift δ_c of an individual control cell, with z_u unchanged. The same cell therefore exists in both states, giving a ground-truth one-to-one pairing (control cell i ↔ treated cell i) that destructive sequencing cannot provide. We form two populations: all cells in the control state, and the same cells in the treated state, and pass them to the conditional OT without any

pairing information, then ask whether the recovered coupling re-pairs each control cell with its true counterpart.

Coupling. For each cell type c (denote sets S_c for control, T_c for treated, where $|S_c| = |T_c| = n_c$), we solve the conditional OT with cost

$$C_{ij} = \alpha \left\| z_{u,i}^s - z_{u,j}^t \right\|_2^2 + \beta \left\| z_{d,i}^s - z_{d,j}^t \right\|_2^2, \quad \alpha = 0.5, \beta = 1,$$

using the exact EMD solver, and row-normalise the plan so that $\sum_j \Gamma_{ij} = 1$ (barycentric weights). Matching is restricted within each cell type. In this paired setting the two populations have equal size, so the exact-EMD plan is a one-to-one permutation; in practice the control and treated populations differ in size (or an entropic/Sinkhorn solver is used), so the coupling is soft, i.e., each control cell is matched to a transport-weighted set of treated cells, and the matched score defined below is the barycentric average over that set.

Response rank. The perturbation acts along the known shift direction $\hat{d}_c = \delta_c / \|\delta_c\|$. Each cell's response score is the projection of its ground-truth z_d onto this axis, $g_i = \langle z_{d,i}^{\text{true}}, \hat{d}_c \rangle$, and its rank r_i is the percentile rank of g_i within its cell type. For a control cell i, the OT-matched score is the barycentric average $\hat{g}_i = \sum_{j \in T_c} \Gamma_{ij}\ g_j$, with matched rank $\hat{r}_i$ the percentile rank of $\hat{g}_i$.

Metrics (per cell type).

We use the Spearman rank correlation and top-1 index

$$\rho_c = \text{Spearman}(\{r_i\}, \{\hat{r}_i\}), \qquad \text{top-1}_c = \frac{1}{n_c} \sum_i \mathbf{1} \left[\arg\max_j \Gamma_{ij} = i \right],$$

i.e. ρ_c measures whether a control cell is matched to a treated cell of the same response rank, and top-1_c the fraction of control cells whose highest-mass partner is their true counterpart i. Both are computed (i) on the ground-truth latent factors (we simulate $dim(z_d) = dim(z_u) = 10$) and (ii) on the factors inferred by the trained scDRP model (we set $dim(z_d) = dim(z_u) = 20$ with L0 penalty strength 0.01); in both cases the ranks $r_i, \hat{r}_i$ use the ground-truth z_d so that the reference ordering is exact and only the coupling Γ changes.

Results. Shown in Table S1, on the ground-truth factors the within-cell-type coupling is the identity matrix (Fig. S4a) and rank preservation is perfect in all cell types (ρ = 1.00, top-1 = 1.00) — the unique optimum under a squared-Euclidean cost and a constant shift. On the model-inferred factors the correspondence is substantially preserved: mean per-cell-type ρ = 0.84 and mean top-1 partner recovery = 0.85, with the coupling remaining concentrated on the diagonal (Fig. S4b). The degree of preservation varies across cell types, reflecting how faithfully each cell type's latent structure is recovered by the encoder. Together these results

show that scDRP resolves the individual control-to-treatment correspondence, both in principle and through the learned representation.

Table S1. Per-cell-type matching on simulated data. Spearman rank correlation (ρ) and top-1 partner recovery, on the ground-truth and model-inferred latent factors.

cell type	n	ρ (true)	top-1 (true)	ρ (learned)	top-1 (learned)
0	230	1.00	1.00	0.78	0.88
1	264	1.00	1.00	0.63	0.60
2	200	1.00	1.00	0.88	0.84
3	244	1.00	1.00	0.97	0.99
4	312	1.00	1.00	0.96	0.95
**mean**	—	**1.00**	**1.00**	**0.84**	**0.85**

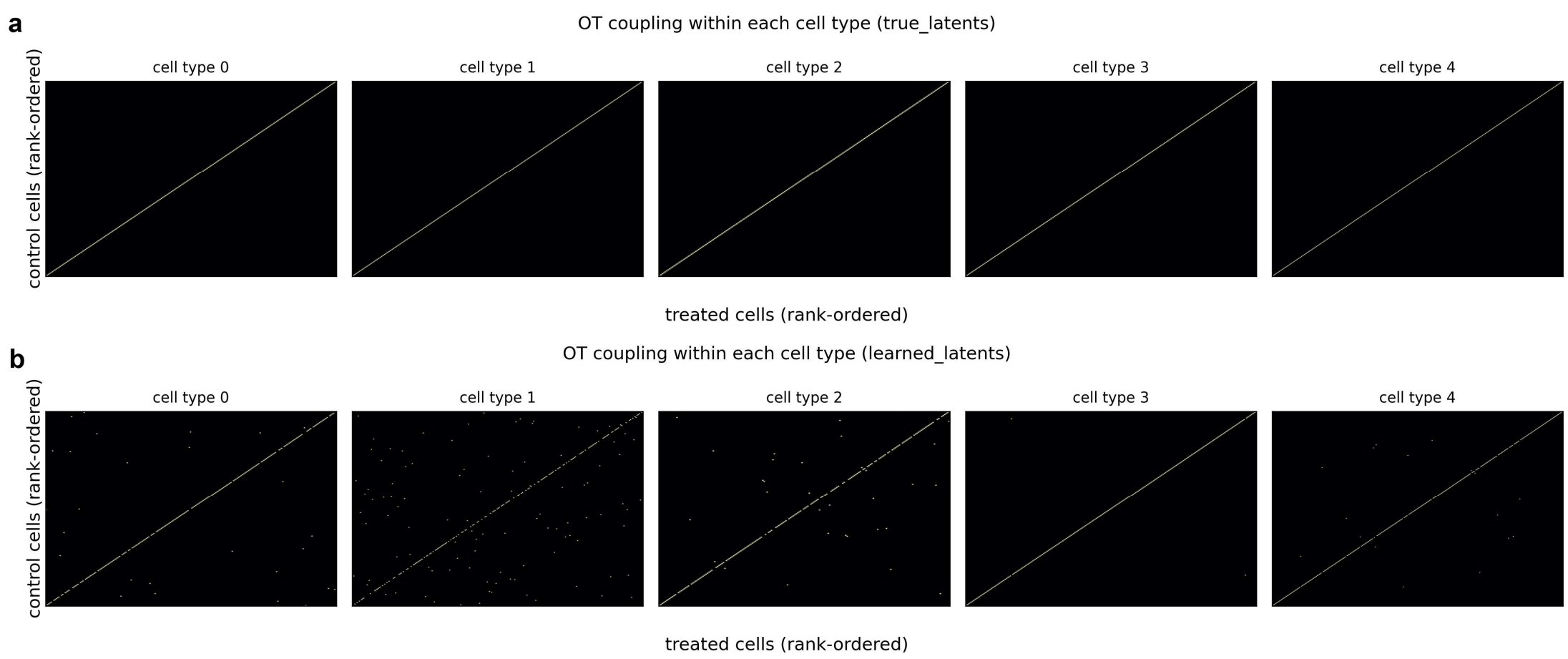

Supplementary Fig. S4. Within-cell-type OT coupling Γ (control rows vs treated columns, both ordered by response rank; one panel per cell type) on (**a**) the true latent factors in data generating process and (**b**) the model-inferred latent factors. A bright entry at position (m, m) indicates that the control cell of rank m is matched to the treated cell of the same rank; the coupling is the identity in (a) and remains concentrated on the diagonal in (b).

SN5 Visualization on learned latent representations from real dataset

To evaluate the efficacy of the learned disentangled latent representations, we performed UMAP visualizations on the Kang dataset for the perturbation-induced latent space (z_d), the perturbation-invariant latent space (z_u), and the full latent representation (z), across two experimental settings: with and without the provision of cell-type labels.

It can be observed that the perturbation-invariant space z_u effectively isolates perturbation-invariant, intrinsic cellular characteristics. Specifically, cells from the treatment and control groups are integrated together, whereas different cell types are distinctly clustered (Fig. S5a). Notably, this precise clustering of cell types is maintained even when cell-type labels are not provided in the training process (Fig. S6a). In contrast, the perturbation-induced space z_d segregates the treatment group from the control group, while simultaneously distinguishing between cell types due to their heterogeneous cell-type specific perturbation responses (Fig. S5b, S6b). When visualizing the full latent space, the UMAP projections clearly indicate that both cell types and experimental conditions are well-resolved, characterized by two separate clusters (treatment and control) for each individual cell type (Fig. S5c, S6c).

Consequently, these results validate that our disentangled representation learning method accurately captures and decouples the latent features underlying single-cell perturbation data, which are subsequently utilized in conditional OT to achieve precise causal effect identification and perturbation response prediction.

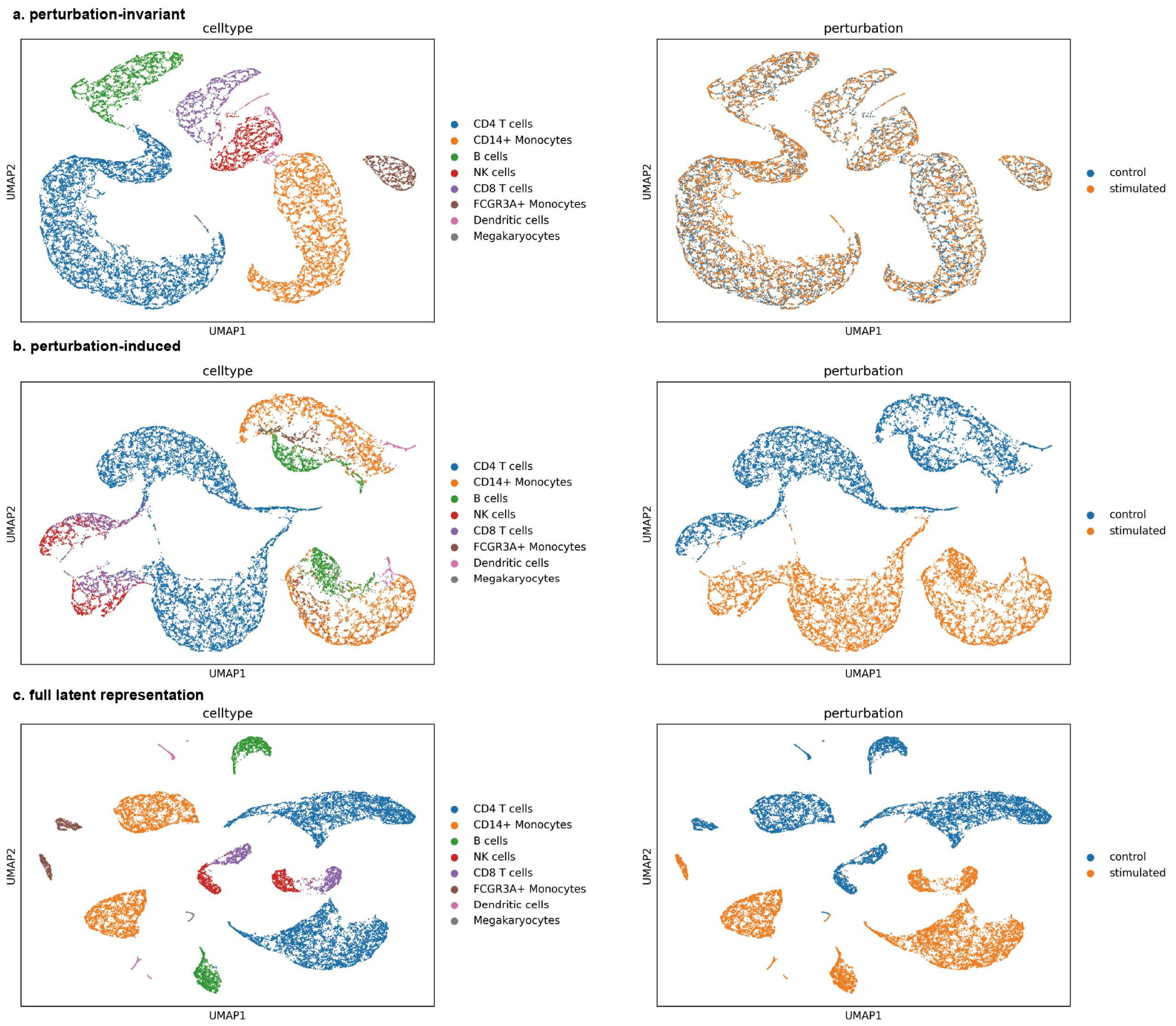

Fig. S5 UMAP visualization of learned latent perturbation-invariant (z_u, a), perturbation-induced (z_d, b) and full latent representation (z, c) for Kang dataset, where the original cell-type labels are explicitly provided in the training process.

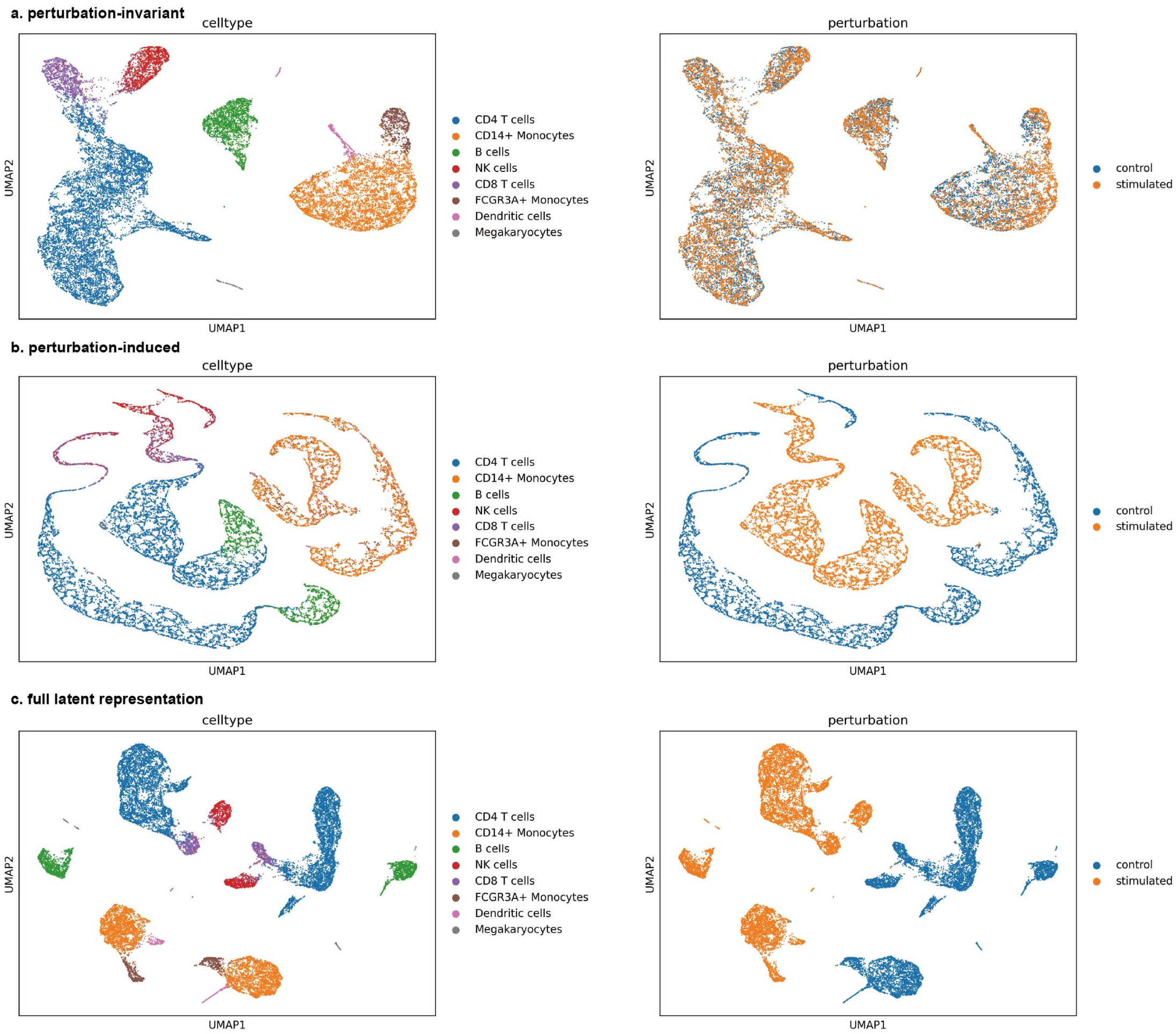

Fig. S6 UMAP visualization of learned latent perturbation-invariant (z_u, a), perturbation-induced (z_d, b) and full latent representation (z, c) for Kang dataset, where the original cell-type labels are not provided in the training process. We can see the representation learning process can still recover the accurate cell identity structures.

Supplementary Figures S7-S18

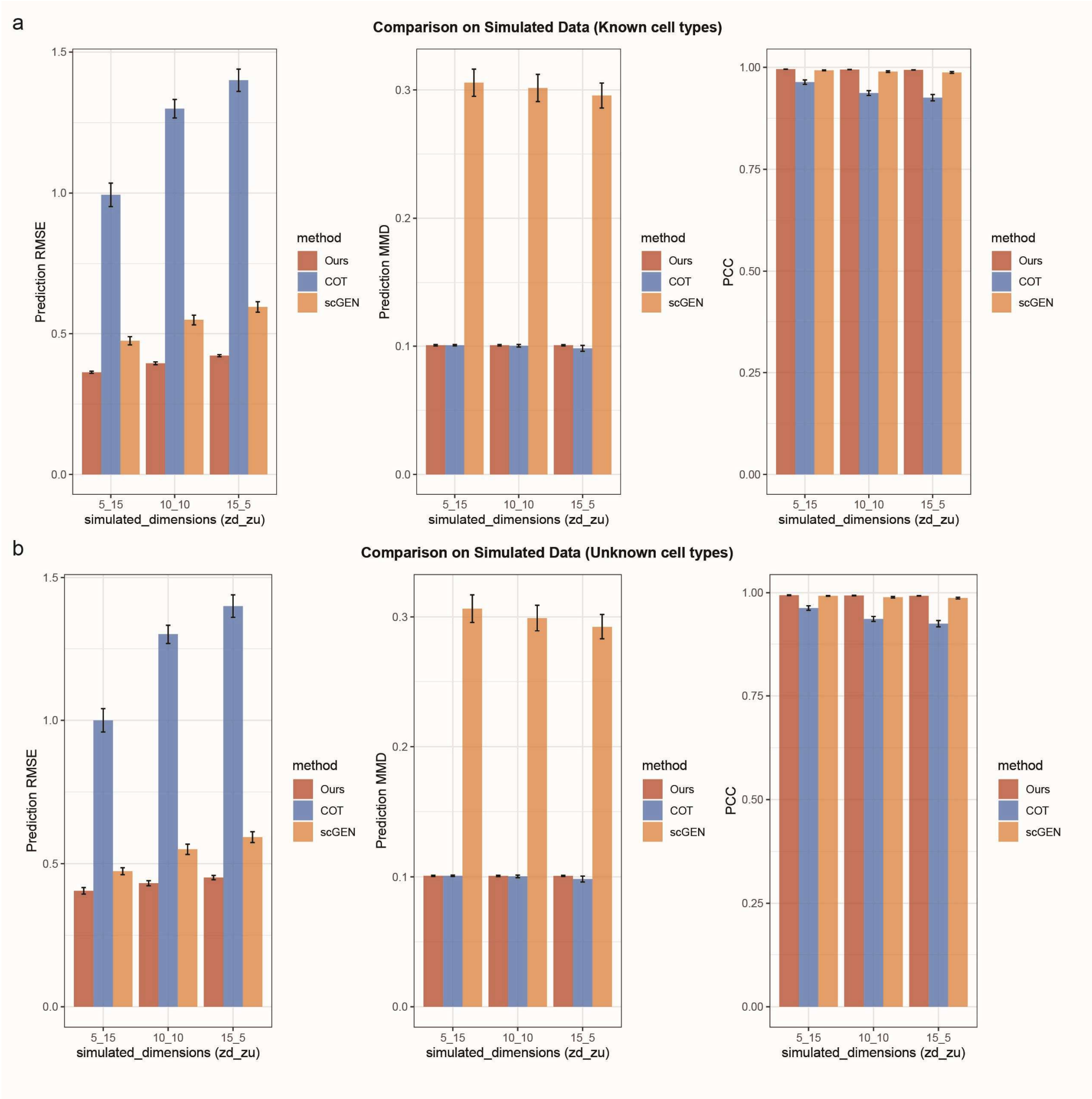

Supplementary Fig. S7 Performance on counterfactual predictions with simulated data under known cell-type labels (a) and unknown cell-type labels (b) settings. We evaluated the RMSE and Pearson correlation coefficient (PCC) of the mean expression of each gene between observed treatment group and counterfactual predictions from control group, as well as the maximum mean discrepancy (MMD) between the observed and predicted distributions. All metrics are averaged across all perturbations and cell types in each simulation. The x-axis shows the dimension settings for z_d and z_u in the simulation, and the different colors correspond to different methods. We repeat each simulated setting 20 times and report the mean$\pm$1.96SE.

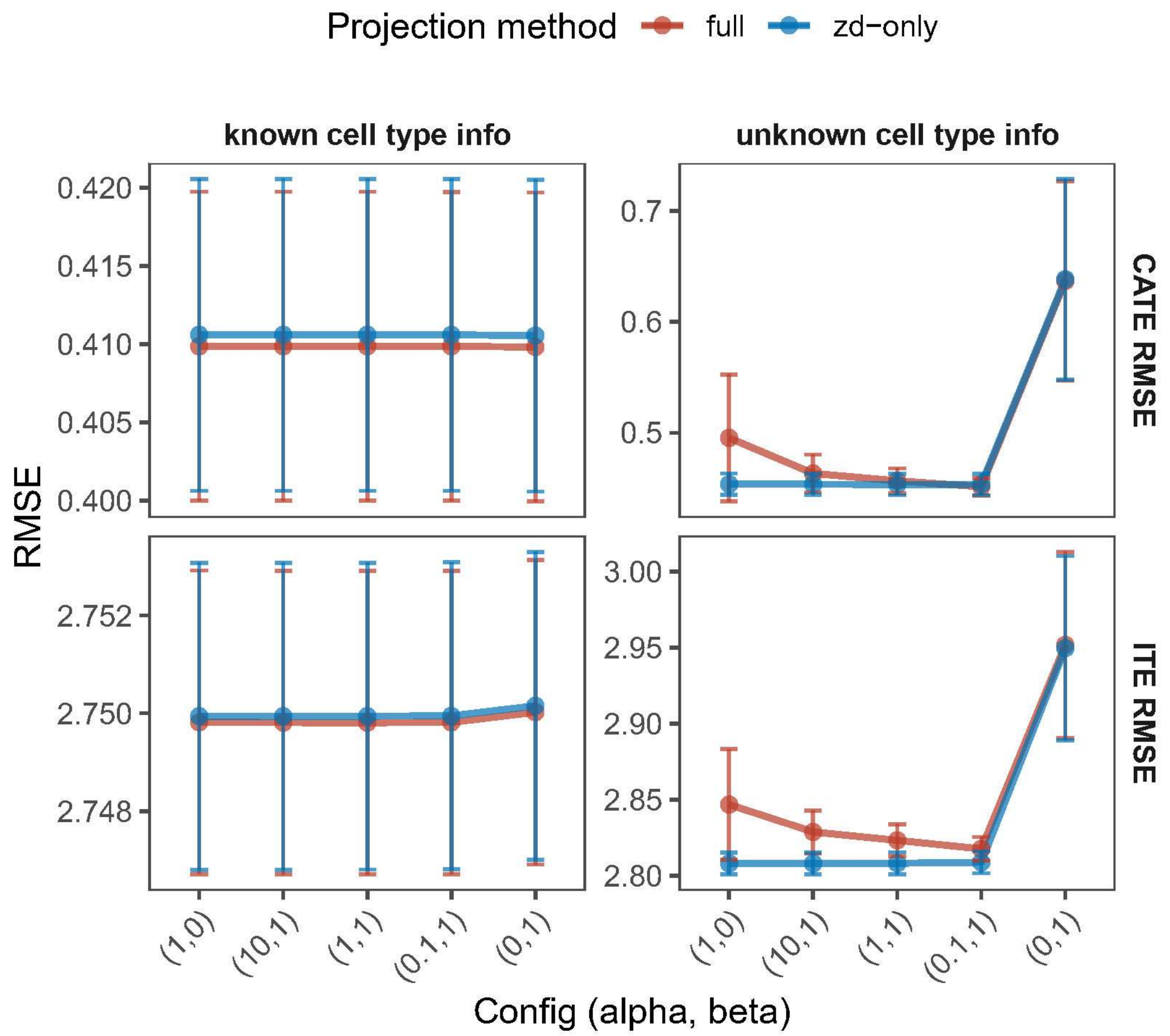

Supplementary Fig. S8 Sensitivity analysis on hyperparameters of conditional OT on simulated data. In each hyperparameter configuration, we repeat each simulated setting 20 times and report the mean RMSE±1.96SE.

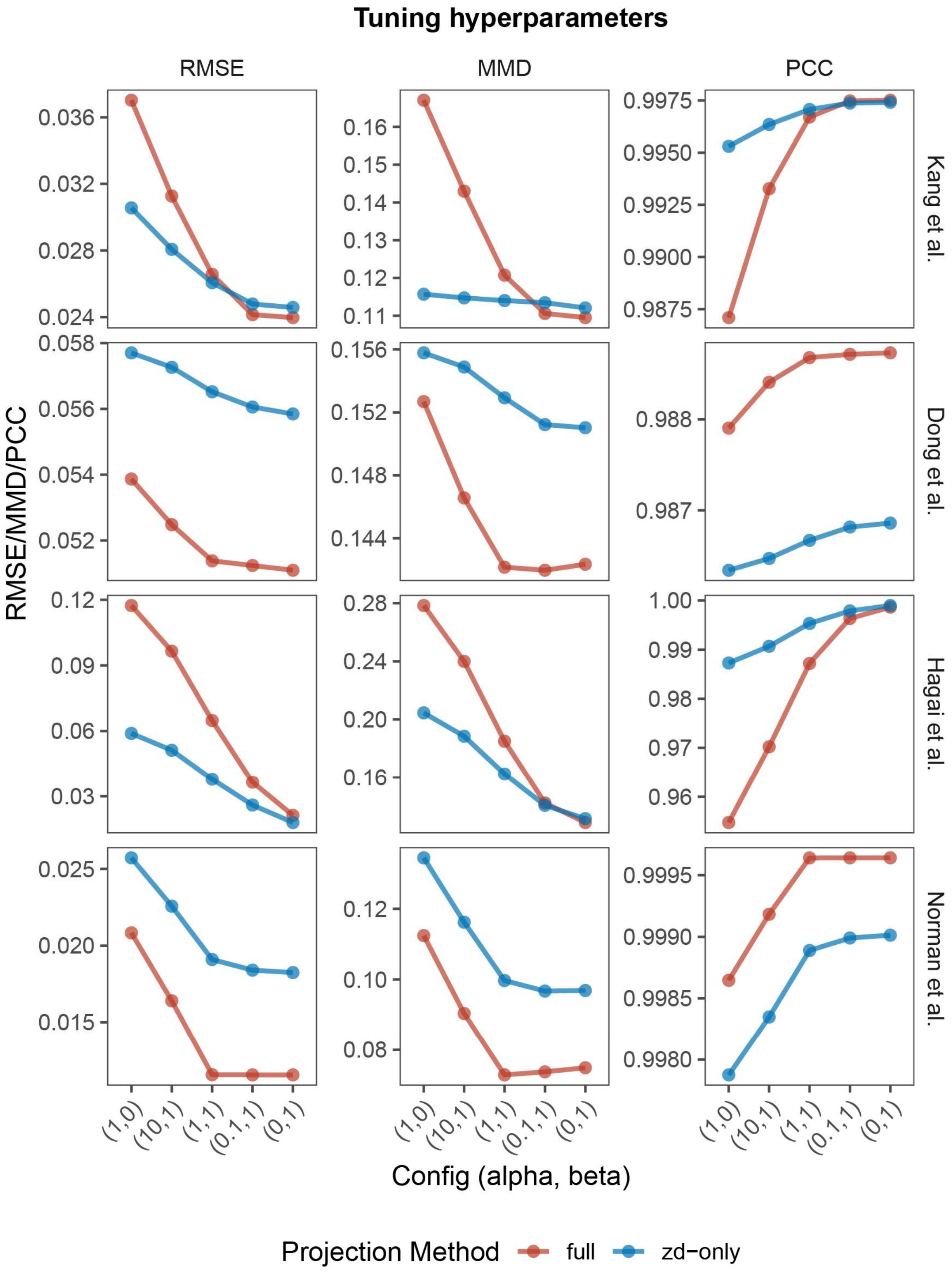

Supplementary Fig. S9 Sensitivity analysis on hyperparameters of conditional OT on real data (known cell-type label).

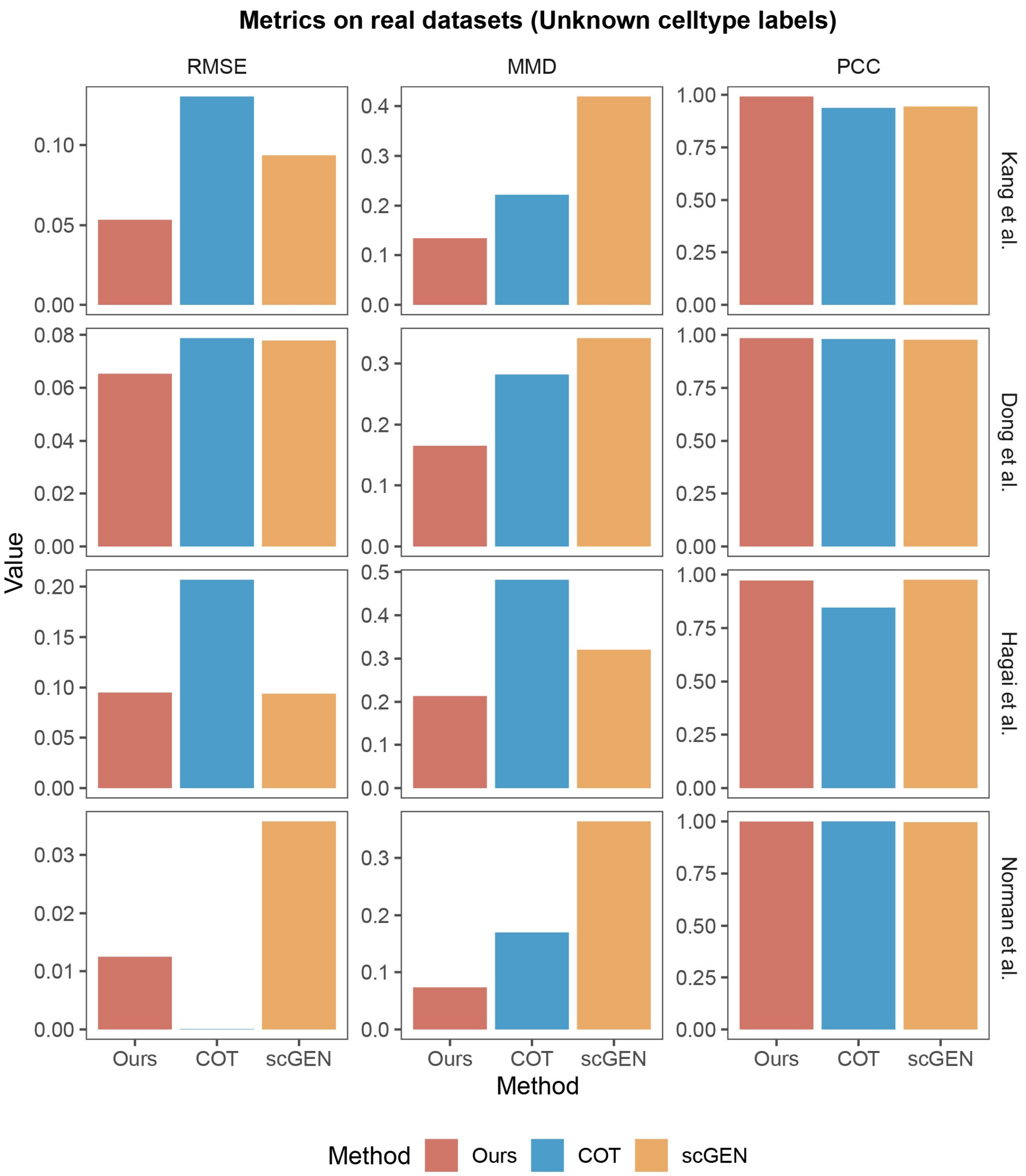

Supplementary Fig. S10 Performance on counterfactual predictions on real single-cell data without cell-type label information provided in training. The evaluation metrics includes RMSE and PCC of the mean expression of each gene between observed treatment group and counterfactual predictions from control group (we compare the mean since the true counterfactual outcome of control cells are not accessible in real data), as well as MMD between the observed and predicted distributions. All metrics are averaged across all perturbations and cell types.

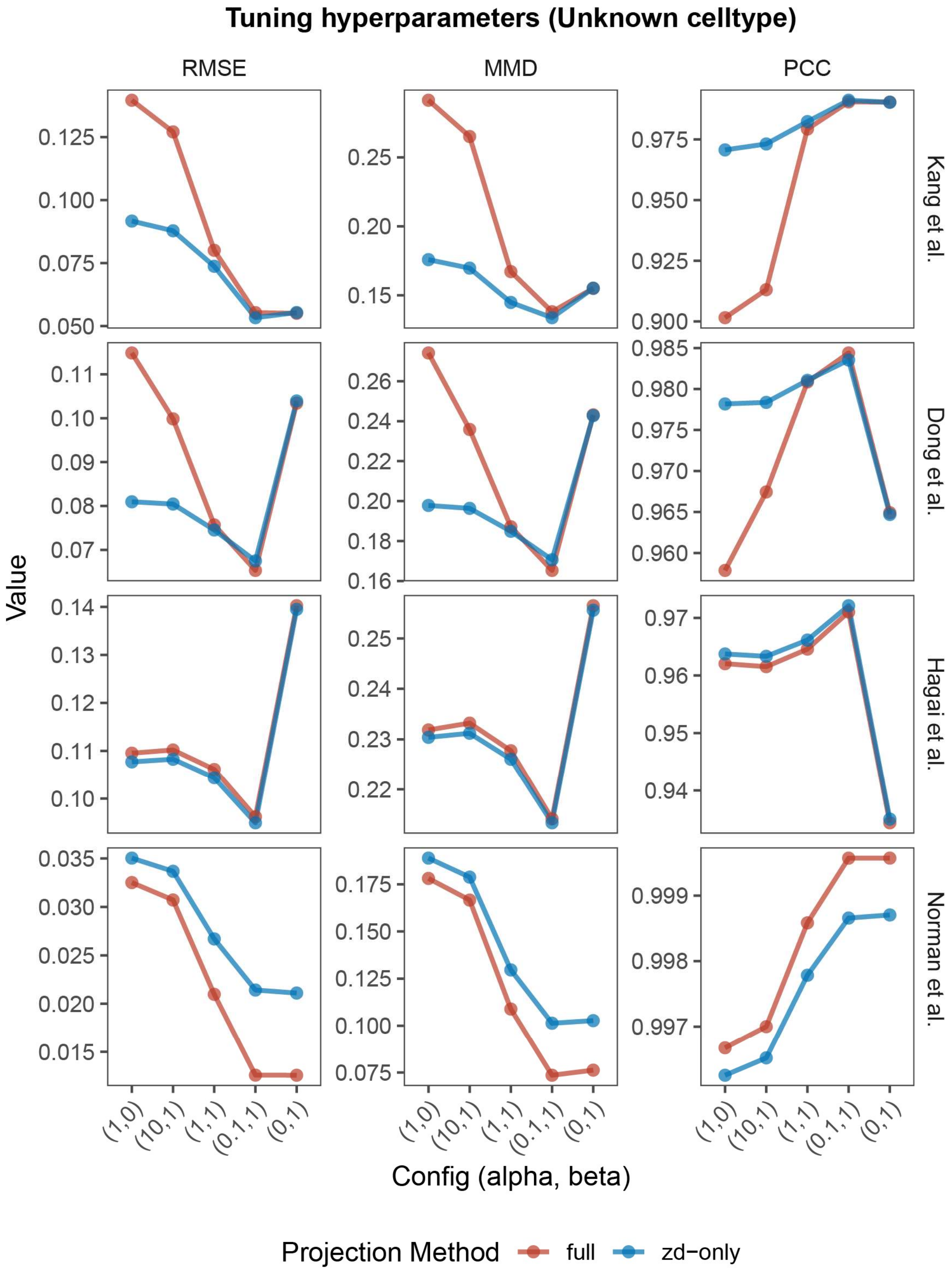

Supplementary Fig. S11 Sensitivity analysis on hyperparameters of conditional OT on real data under the unknown cell-type labels setting.

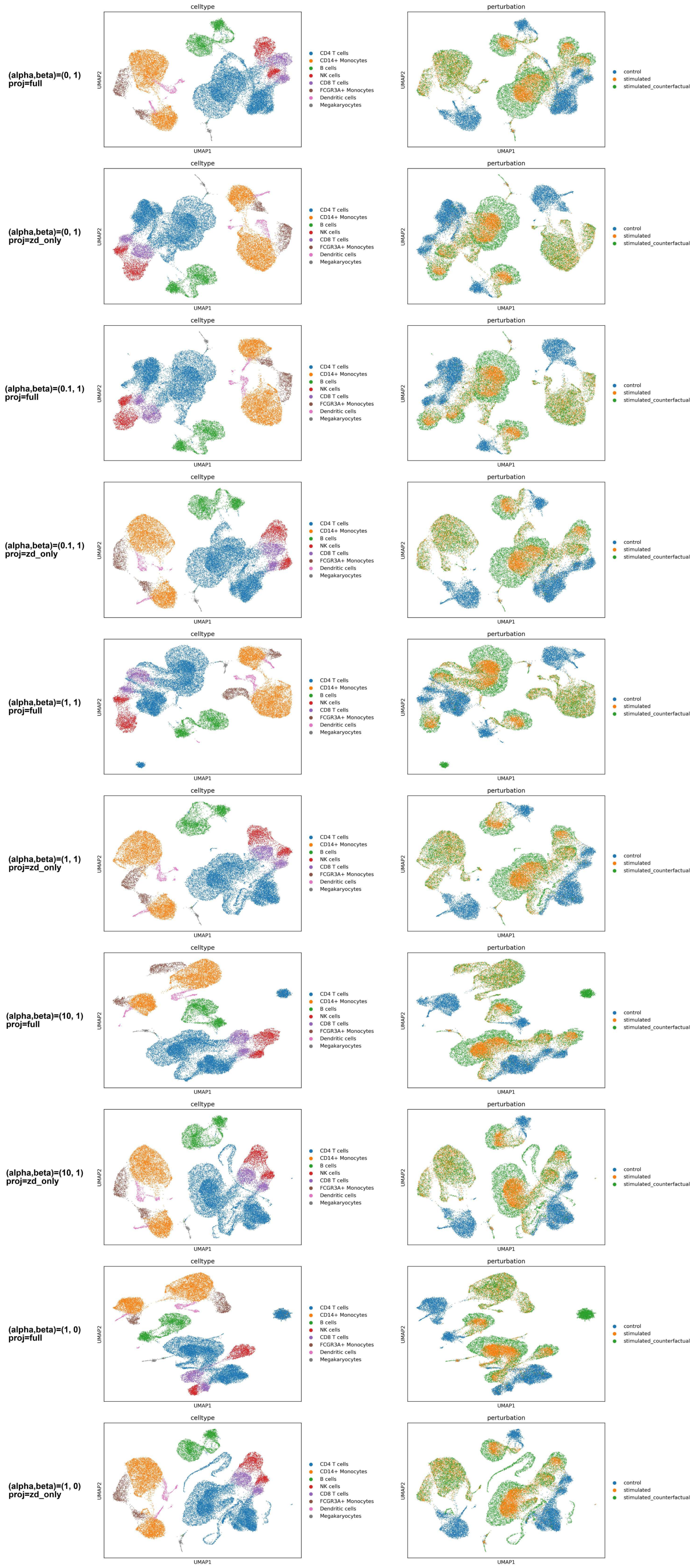

Supplementary Fig. S12 UMAP visualization of counterfactual predictions on Kang dataset under different settings of alpha, beta, and barycentric projection methods (known cell

types).

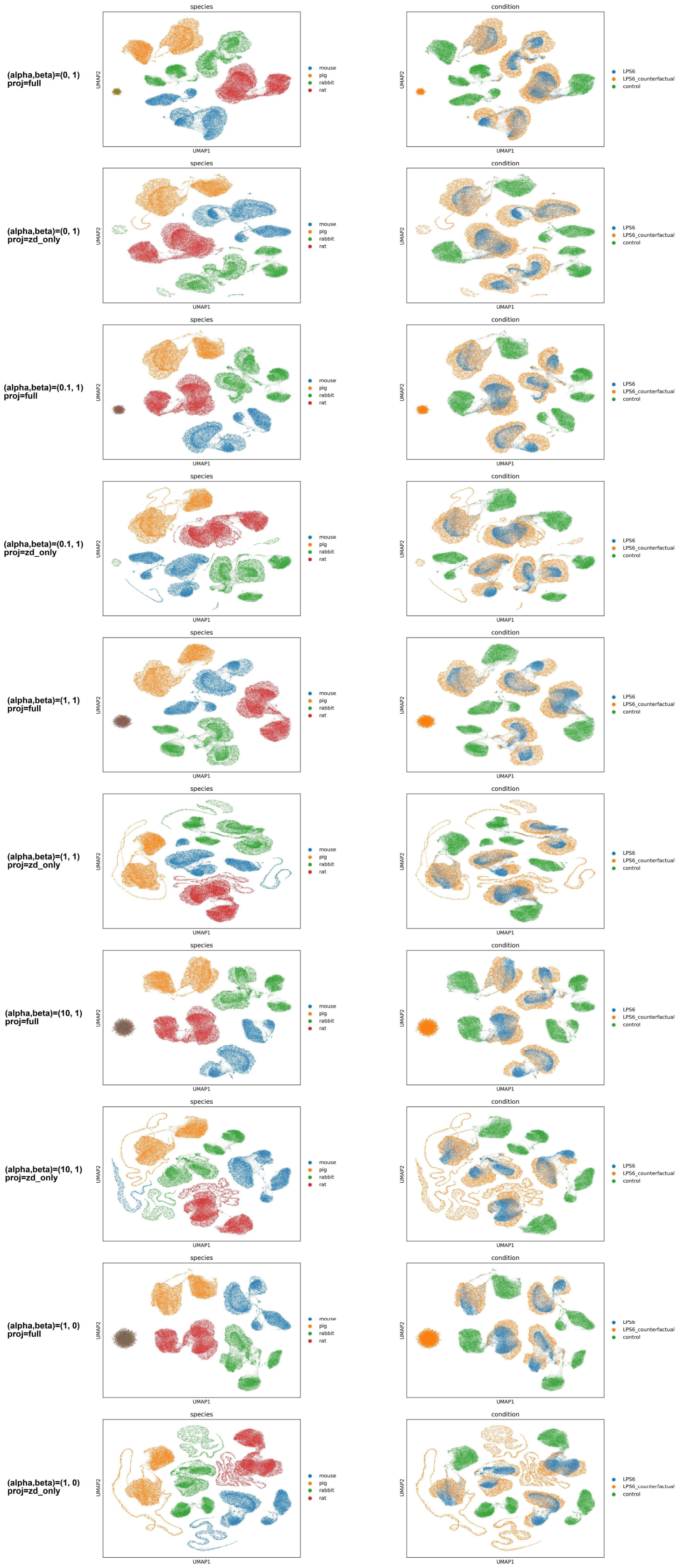

Supplementary Fig. S13 UMAP visualization of counterfactual predictions on Hagai

dataset under different settings of alpha, beta, and barycentric projection methods (known cell types).

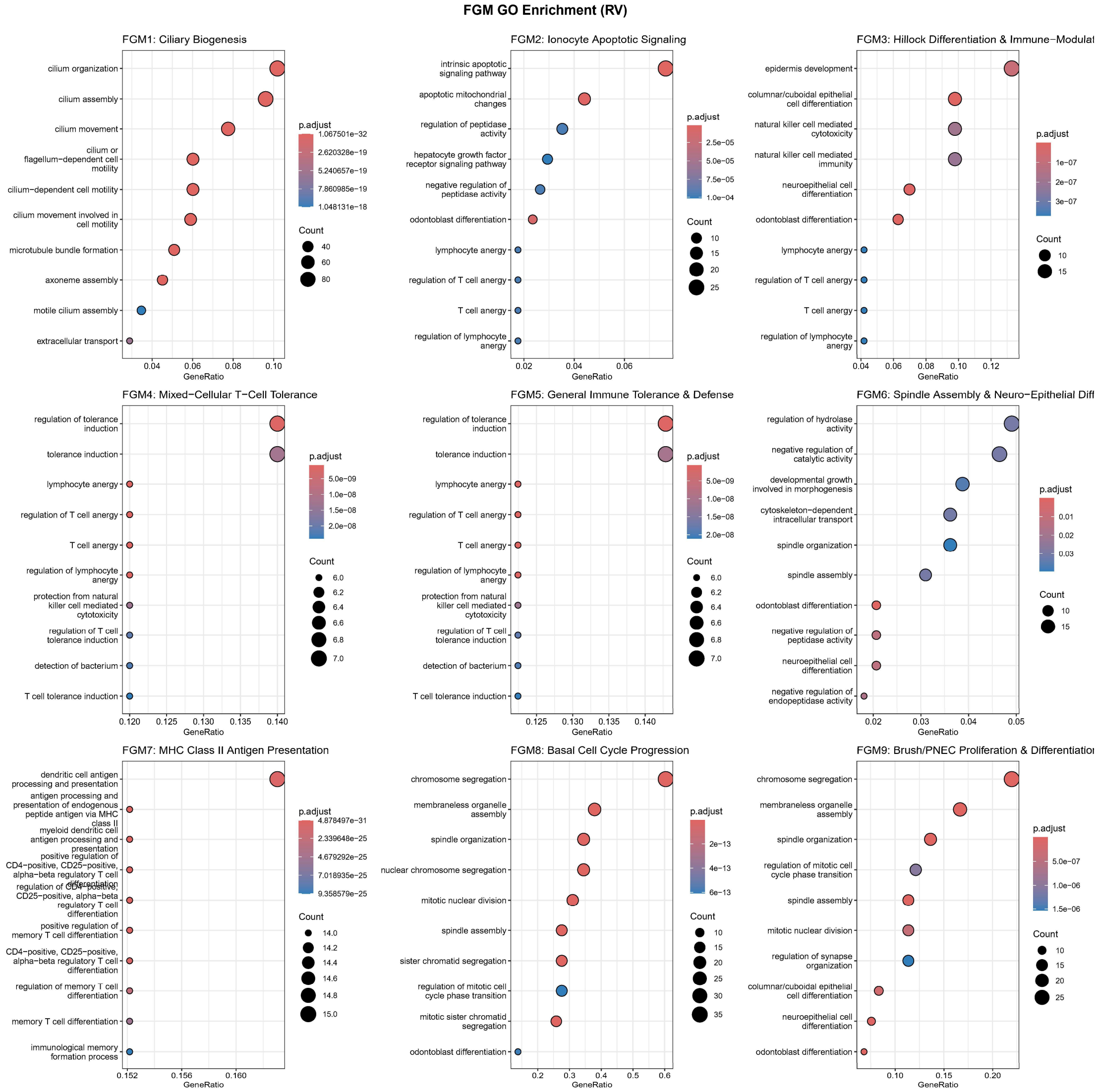

Supplementary Fig. S14 Details on GO enrichment of FGM identified in primary human bronchial organoids under rhinovirus (RV) exposure.

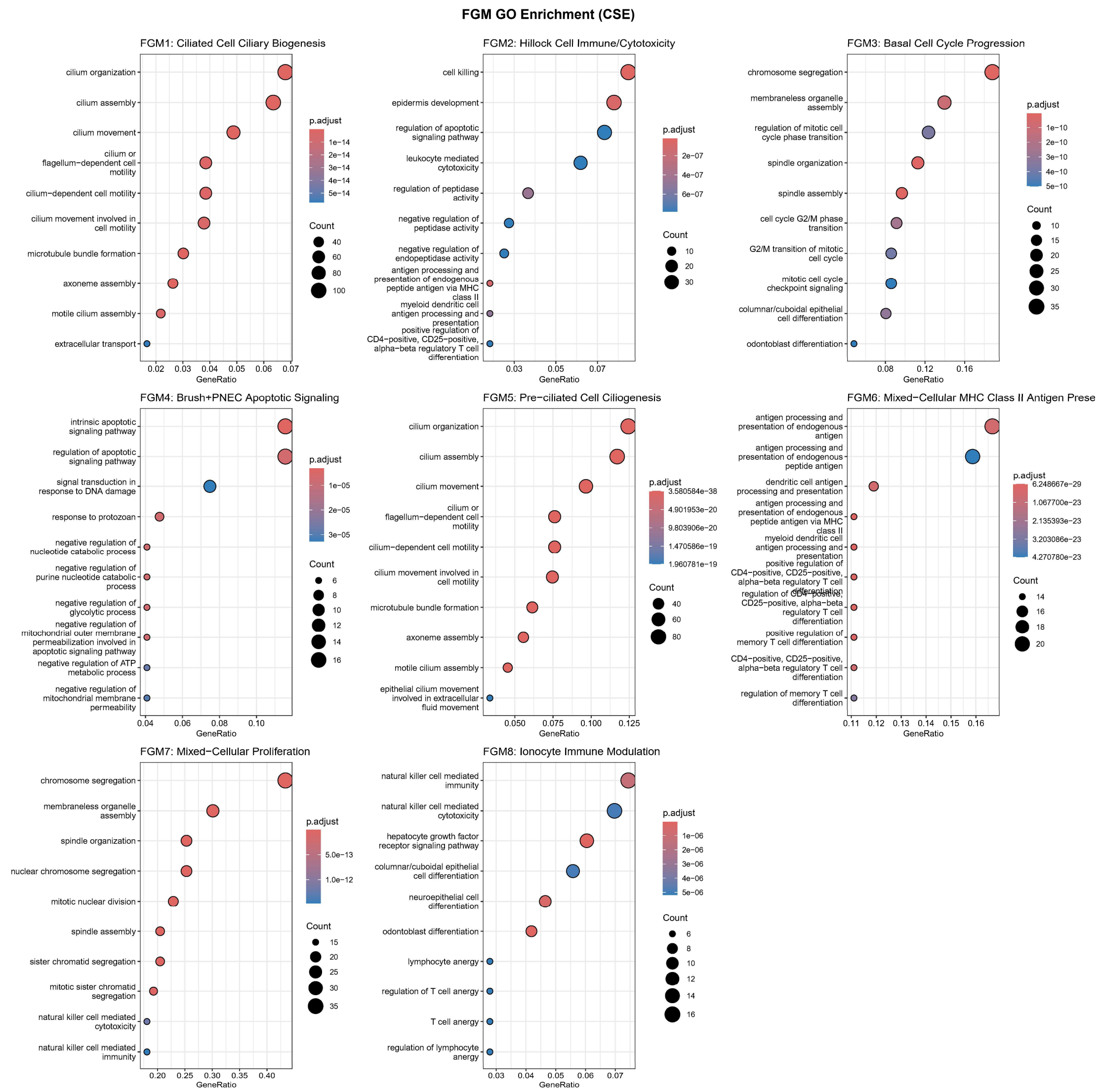

Supplementary Fig. S15 Details on GO enrichment of FGM identified in primary human bronchial organoids under cigarette-smoke extract (CSE) exposure.

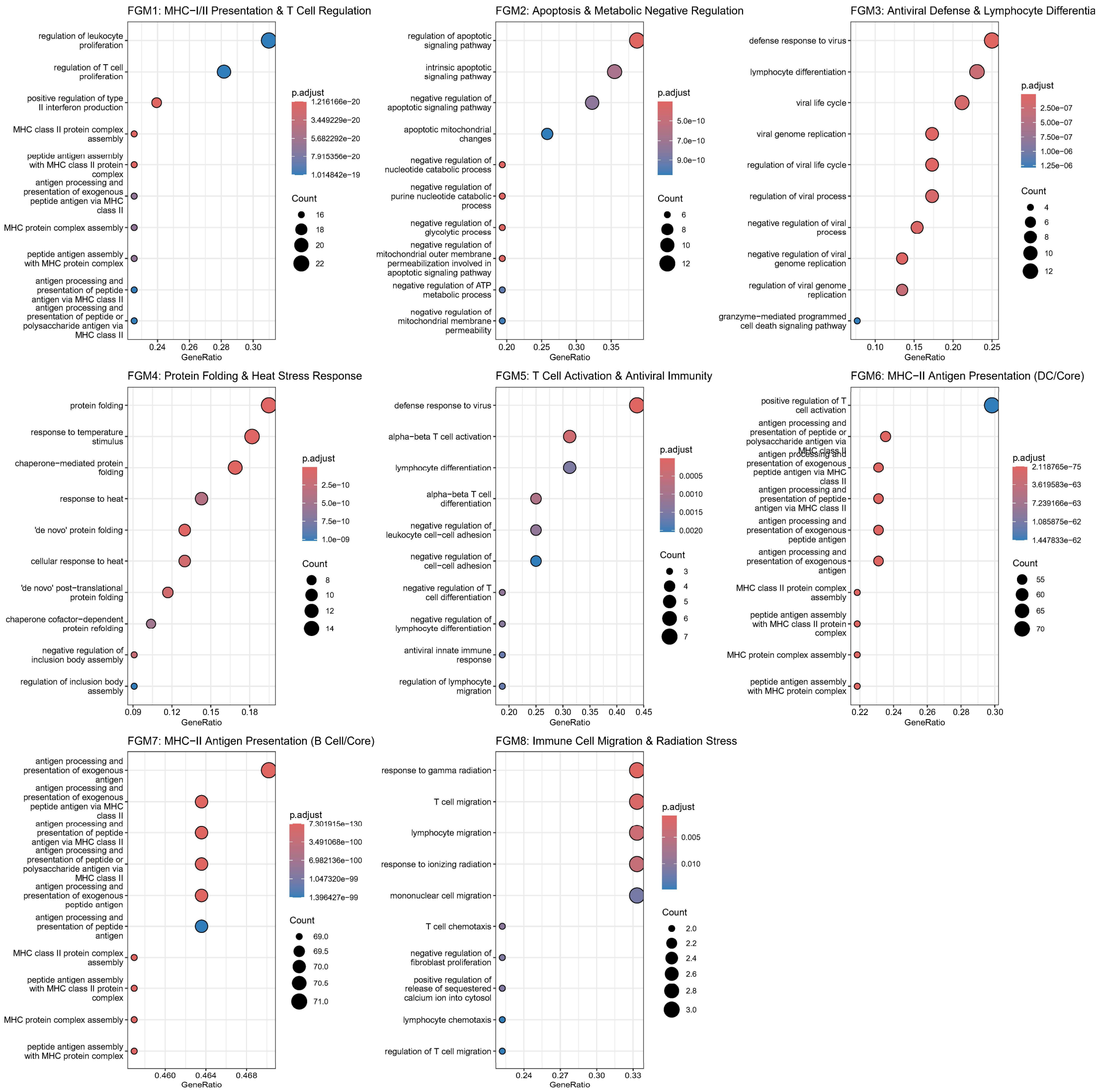

Supplementary Fig. S16   Details on GO enrichment of FGM identified from Kang dataset.

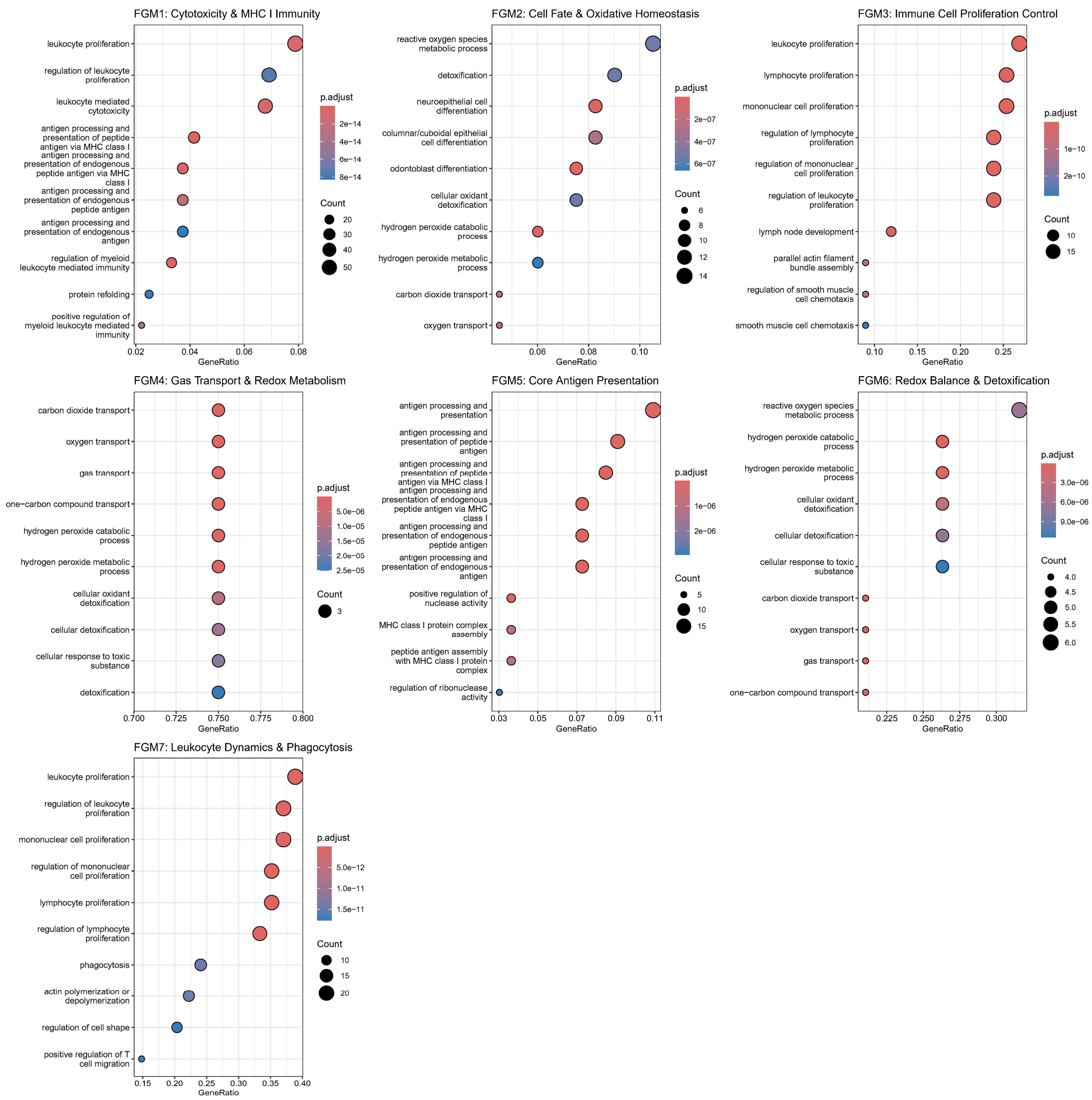

Supplementary Fig. S17 Details on GO enrichment of FGM identified from Norman CRISPR-a Perturb-seq dataset.

Supplementary Note SN6 Nucleosome-related gene effect analysis with CRISPR-a Perturb-Seq

We investigated how CRISPR-a perturbations relate to nucleosome-related and non-nucleosome-related genes using the estimated ITE matrix on the Norman data. We used CpG island (CGI) promoter status as an imperfect proxy for promoter nucleosome architecture: non-CGI promoters tend to be assembled into stable, positioned nucleosomes and to require stimulus-dependent chromatin remodeling for activation, whereas CGI promoters tend to be nucleosome-depleted and constitutively accessible [9,10]. This proxy is grounded in the sequence determinants of nucleosome organization: CpG and GC content correlate with nucleosome depletion at mammalian promoters both *in vivo* and *in vitro* [11], and interpretable sequence-based models trained on MNase-seq data identify CpG-containing patterns among the dominant determinants of nucleosome organization: the CCG/CGG trinucleotide is depleted on nucleosomal relative to inter-nucleosomal DNA, and its periodicity is the top-ranked feature for discriminating nucleosomal from inter-nucleosomal regions [12]. CGI status can therefore be viewed as a coarse, promoter-level summary of the same sequence signal that dictates nucleosome organization at nucleotide resolution.

Each gene was classified by overlapping its promoter region (TSS$\pm$1 kb) with annotated CpG islands, yielding 942 non-CGI and 1,045 CGI genes out of the 3,000 highly variable genes. For each gene we computed its mean absolute ITE (effect magnitude) per perturbation from the inferred ITE matrix, and compared the two promoter classes both unadjusted (Mann–Whitney U test) and controlling for baseline expression (using expression as a covariate, as well as a more robust expression-decile-stratified test), given that both effect magnitude and CGI status correlate with expression level.

The result is an expression-dependent pattern. Without adjustment, CGI genes appear to have larger effects (Cliff's $\delta \approx -0.2$), but this is attributable to their higher baseline expression. After controlling for expression, the relationship reversed and remained consistent across all eight perturbations: non-CGI promoters show larger perturbation-induced transcriptional responses than CGI promoters, with a positive adjusted coefficient in all eight perturbations (expression-decile-stratified $p < 0.01$ for all eight; the expression-covariate model agrees in direction for all eight and reaches $p < 0.05$ for seven of eight) (Fig. S18). This is consistent with previous reports that non-CGI promoters generally display higher nucleosome occupancy and stronger nucleosome positioning, requiring stimulus-dependent chromatin remodeling for activation, whereas CGI promoters tend to adopt nucleosome-depleted and more permissive chromatin architectures [9, 10].

We acknowledge several key limitations of the current analysis. First, the effects, while

statistically robust, are modest in magnitude. Second, CGI status is only an imperfect proxy for nucleosome occupancy: low nucleosome occupancy is a general property of active promoters and non-CGI promoters are heterogeneous. This imperfect proxy may reduce our ability to fully resolve the relationship between promoter chromatin architecture and perturbation responsiveness. Third, sequence-governed nucleosome positioning is statistical in nature and can be overridden *in vivo* by chromatin remodelers, as reflected in the reduced accuracy of sequence-based predictors in transcriptionally active relative to resting cells [12]. Analyses of perturbation effects on directly measured chromatin-level features, such as scATAC-seq data, remain open for future studies.

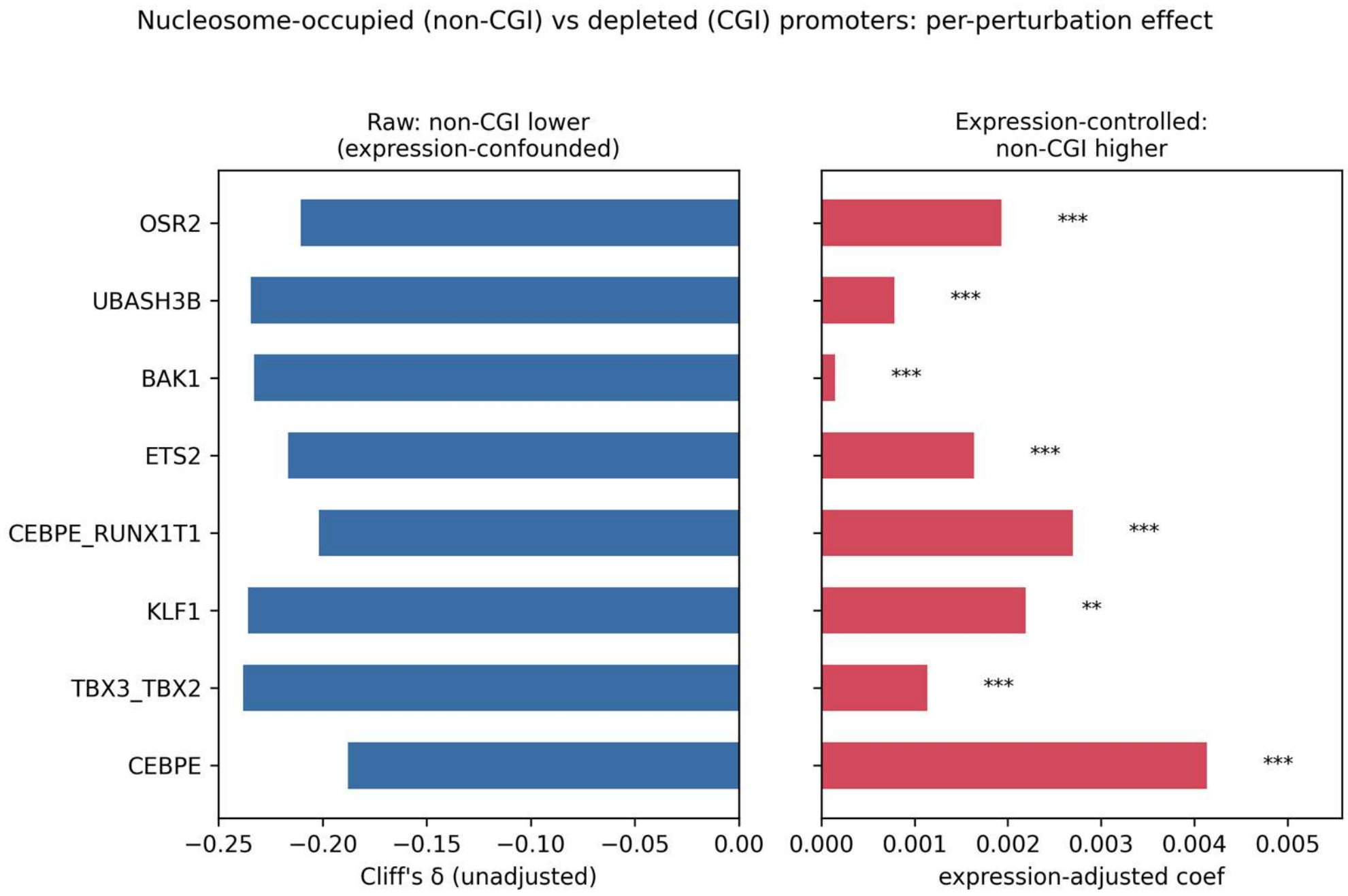

Supplementary Fig. S18. Nucleosome-occupied (non-CGI) promoter genes show larger treatment effects than nucleosome-depleted (CGI) genes after controlling for expression. Per-gene effect magnitude (mean |ITE|) was compared between genes with non-CGI (nucleosome-occupied, $n = 942$) and CGI (nucleosome-depleted, $n = 1{,}045$) promoters across the eight K562 CRISPR-activation perturbations. Left: unadjusted (Cliff's δ); $\delta \approx -0.2$ indicates non-CGI genes appear less responsive, reflecting their lower baseline expression. Right: after controlling for expression, the coefficient is positive in every perturbation: non-CGI genes are more responsive at matched expression (*** $p < 0.001$, ** $p < 0.01$, expression-decile-stratified test). CpG-island status is a proxy for promoter nucleosome architecture.

Reference

[9] Ramirez-Carrozzi, V. R., Braas, D., Bhatt, D. M., Cheng, C. S., Hong, C., Doty, K. R., ... & Smale, S. T. (2009). A unifying model for the selective regulation of inducible transcription

by CpG islands and nucleosome remodeling. *Cell*, *138*(1), 114-128.

[10] Ahmad, K., Henikoff, S., & Ramachandran, S. (2022). Managing the steady state chromatin landscape by nucleosome dynamics. *Annual review of biochemistry*, *91*(1), 183-195.

[11] Fenouil, R., Cauchy, P., Koch, F., Descostes, N., Cabeza, J. Z., Innocenti, C., ... & Andrau, J. C. (2012). CpG islands and GC content dictate nucleosome depletion in a transcription-independent manner at mammalian promoters. *Genome research*, *22*(12), 2399-2408.

[12] Masoudi-Sobhanzadeh, Y., Li, S., Peng, Y., & Panchenko, A. R. (2024). Interpretable deep residual network uncovers nucleosome positioning and associated features. *Nucleic Acids Research*, *52*(15), 8734-8745.

Supplementary Tables S2-S5

Supplementary Table S2 Details on the FGM identified in primary human bronchial organoids under rhinovirus (RV) exposure.

FGM ID	Module Name	Functional Description
**FGM1**	Ciliary Biogenesis Module	This module is overwhelmingly dominated by Pre-ciliated cells and Doublets. Its functional signature is characterized by an exceptionally strong enrichment for pathways related to ciliary construction, including 'cilium organization', 'cilium assembly', 'axoneme assembly', and 'motile cilium assembly'. This reflects a highly active program of ciliogenesis localized to this specific cell population.
**FGM2**	Ionocyte Apoptotic Signaling Module	A highly specific module composed almost exclusively of Ionocytes. It is functionally defined by the 'intrinsic apoptotic signaling pathway' and 'apoptotic mitochondrial changes', indicating a distinct, cell-type-specific apoptotic response to the RV treatment.
**FGM3**	Hillock Differentiation & Immune-Modulatory Module	This module is driven by Hillock cells and exhibits a dual function. It is enriched for epithelial development terms like 'columnar/cuboidal epithelial cell differentiation' while concurrently showing a strong signature for innate and adaptive immune modulation, including 'natural killer cell mediated cytotoxicity' and 'regulation of T cell anergy'.
**FGM4**	Mixed-Cellular T-Cell Tolerance Module	A compositionally heterogeneous module with contributions from Ciliated, Basal, and Secretory cells. Functionally, it shows a highly coherent signature for immune suppression, strongly enriched for 'regulation of tolerance induction', 'T cell anergy', and 'T cell tolerance induction'.
**FGM5**	General Immune Tolerance & Defense Module	Similar to FGM4, this is another mixed-cellularity module defined by immune tolerance pathways such as 'tolerance induction' and 'regulation of T cell anergy'. It uniquely includes terms for 'protection from natural killer cell mediated cytotoxicity', suggesting a role in both modulating and evading immune responses.

**FGM6**	Spindle Assembly & Neuro-Epithelial Differentiation Module	This mixed module, with notable contributions from Secretory and Basal cells, is linked to cell division and differentiation. It is significantly enriched for 'spindle organization' and 'spindle assembly', alongside specialized differentiation terms 'odontoblast differentiation' and 'neuroepithelial cell differentiation'.
**FGM7**	MHC Class II Antigen Presentation Module	A key immune response module with broad cellular contribution, primarily from Secretory, Basal, and Ciliated cells. Its function is unambiguously centered on adaptive immunity, defined by enrichment for 'dendritic cell antigen processing and presentation' and 'antigen processing and presentation of endogenous peptide antigen via MHC class II'.
**FGM8**	Basal Cell Cycle Progression Module	This module is almost entirely composed of 'Cycling basal' cells and represents a canonical cell cycle program. It is strongly enriched for all phases of mitosis, including 'chromosome segregation', 'spindle organization', 'mitotic nuclear division', and 'mitotic sister chromatid segregation', reflecting active proliferation of the basal stem cell compartment.
**FGM9**	Brush/PNEC Proliferation & Differentiation Module	Driven almost exclusively by the Brush+PNEC cell population, this module captures a proliferative response within this rare cell type. It is highly enriched for cell cycle terms like 'chromosome segregation' and 'spindle organization', but also includes epithelial differentiation terms, suggesting a coupled proliferation and differentiation program.

Supplementary Table S3 Details on the FGM identified in primary human bronchial organoids under cigarette-smoke extract (CSE) exposure.

FGM ID	Module Name	Functional Description
**FGM1**	Ciliated Cell Ciliary Biogenesis Module	This module is predominantly composed of Ciliated cells. Its functional signature is a strong enrichment for pathways related to ciliary structure and function, including 'cilium organization', 'cilium assembly', 'cilium movement', and 'motile cilium assembly'.

**FGM2**	Hillock Cell Immune/Cytotoxicity Module	This module is highly specific to Hillock cells. It is functionally defined by immune response pathways, notably 'leukocyte mediated cytotoxicity' and 'regulation of peptidase activity', suggesting a role in cell-mediated defense and protein regulation under CSE stress.
**FGM3**	Basal Cell Cycle Progression Module	Driven by Basal and Cycling basal cells, this module represents a classic proliferative response. It is strongly enriched for all phases of mitosis, including 'chromosome segregation', 'spindle organization', and 'cell cycle G2/M phase transition'.
**FGM4**	Brush+PNEC Apoptotic Signaling Module	This module is uniquely dominated by the Brush+PNEC cell population. Its GO terms are tightly focused on 'intrinsic apoptotic signaling pathway' and 'regulation of apoptotic signaling pathway', indicating a specific pro-apoptotic ITE in this cell type.
**FGM5**	Pre-ciliated Cell Ciliogenesis Module	This module is almost exclusively composed of Pre-ciliated cells. Similar to FGM1 but in a precursor population, it is overwhelmingly enriched for the machinery of ciliary construction, including 'cilium organization', 'cilium assembly', and 'axoneme assembly'.
**FGM6**	Mixed-Cellular MHC Class II Antigen Presentation Module	This is a heterogeneous module with contributions from Basal, Ciliated, and Secretory cells. Its function is clearly defined by adaptive immunity, showing strong enrichment for 'antigen processing and presentation of endogenous peptide antigen via MHC class II' and 'dendritic cell antigen processing and presentation'.
**FGM7**	Mixed-Cellular Proliferation Module	A compositionally mixed module, with inputs from Cycling basal and Secretory cells. Like FGM3, it is defined by cell cycle progression, with enrichment for 'chromosome segregation', 'spindle organization', and 'spindle assembly', suggesting a proliferative response outside the main basal compartment.
**FGM8**	Ionocyte Immune Modulation Module	This module is driven by Ionocytes and is characterized by immune modulation pathways. It is enriched for

		'natural killer cell mediated cytotoxicity', 'regulation of T cell anergy', and 'lymphocyte anergy'.

Supplementary Table S4 Details on the FGM identified in Kang dataset.

FGM NO.	SHORT NAME	DETAILED FUNCTIONAL DESCRIPTION (GO ENRICHMENT SUMMARY)
**FGM 1**	MHC I/II Presentation & T Cell Regulation	Highly enriched in MHC I/II antigen processing and presentation (both classes) and the Regulation of leukocyte and T cell proliferation. Also includes positive regulation of Type II IFN production.
**FGM 2**	Apoptosis & Metabolic Negative Regulation	Centered on the Regulation of apoptotic signaling pathway and Negative regulation of metabolic processes, including purine nucleotide catabolism and glycolysis, often via mitochondrial pathways.
**FGM 3**	Antiviral Defense & Lymphocyte Differentiation	Core function is the Defense response to virus and Lymphocyte differentiation. Key features include Viral genome replication regulation and Granzyme-mediated programmed cell death signaling.
**FGM 4**	Protein Folding & Heat Stress Response	Highly specialized for Protein folding (‘de novo’, chaperone-mediated) and the Response to heat or temperature stimulus. Reflects the cellular machinery maintaining proteostasis.
**FGM 5**	T Cell Activation & Antiviral Immunity	Characterized by a strong Defense response to virus and the Alpha–beta T cell activation and differentiation, including negative regulatory aspects of these processes.
**FGM 6**	MHC II Antigen Presentation (DC/Core)	Highly specific for the Antigen processing and presentation of exogenous peptide via MHC class II. Also includes MHC class II protein complex assembly and Positive regulation of T cell activation.
**FGM 7**	MHC II Antigen Presentation	Functional profile is nearly identical to FGM6, focusing on Antigen processing and presentation of exogenous antigen via

	(B Cell/Core)	MHC class II, confirming this as a central APC response.
**FGM 8**	Immune Cell Migration & Radiation Stress	Defined by Mononuclear/Lymphocyte/T cell migration and chemotaxis. Also includes the Response to gamma and ionizing radiation and Positive regulation of calcium ion release.

Supplementary Table S5 Details on the FGM identified in Norman dataset.

FGM ID	SHORT NAME	DETAILED FUNCTIONAL DESCRIPTION (GO TERMS)
**FGM1**	Cytotoxicity & MHC I Immunity	Highly concentrated on leukocyte mediated cytotoxicity, MHC class I antigen processing and presentation (of peptide and endogenous antigen), and leukocyte proliferation. This module is a core driver of CD8+ T cell and NK cell immune responses.
**FGM2**	Cell Fate & Oxidative Homeostasis	Involved in specific cell differentiation processes (e.g., odontoblast, neuroepithelial cell differentiation) and the regulation of reactive oxygen species metabolic process, hydrogen peroxide catabolism, and general detoxification.
**FGM3**	Immune Cell Proliferation Control	Focused entirely on the positive and negative regulation of leukocyte, lymphocyte, and mononuclear cell proliferation. It also includes lymph node development. This module controls the numerical expansion of immune cells during a response.
**FGM4**	Gas Transport & Redox Metabolism	Primary functions include carbon dioxide transport, oxygen transport, and one-carbon compound transport. Also heavily involved in cellular oxidant detoxification and hydrogen peroxide catabolic process, maintaining the cell's metabolic and redox stability.
**FGM5**	Core Antigen Presentation	Highly specific for the machinery of the MHC I pathway: antigen processing and presentation (via MHC I), MHC class I protein complex assembly, and peptide antigen assembly. This is a fundamental component of cell-mediated immunity.
**FGM6**	Redox Balance &	A metabolic and protective module focused on ROS and H2 O2 catabolism, cellular oxidant detoxification, and the cellular response to toxic substances. Also includes gas transport

	Detoxification	functions.
**FGM7**	Leukocyte Dynamics & Phagocytosis	Functions related to leukocyte/lymphocyte proliferation, phagocytosis, T cell migration, and actin polymerization/cell shape regulation. This module governs the rapid growth, movement, and particle-clearing ability of immune cells.
